# Vital Signs: Variation Among States in Prescribing of Opioid Pain Relievers and Benzodiazepines — United States, 2012

**Published:** 2014-07-04

**Authors:** Leonard J. Paulozzi, Karin A. Mack, Jason M. Hockenberry

**Affiliations:** 1 Division of Unintentional Injury Prevention, National Center for Injury Prevention and Control, CDC; 2 Division of Analysis, Research, and Practice Integration, National Center for Injury Prevention and Control, CDC; 3 Department of Health Policy and Management, Rollins School of Public Health, Emory University

## Abstract

**Background:**

Overprescribing of opioid pain relievers (OPR) can result in multiple adverse health outcomes, including fatal overdoses. Interstate variation in rates of prescribing OPR and other prescription drugs prone to abuse, such as benzodiazepines, might indicate areas where prescribing patterns need further evaluation.

**Methods:**

CDC analyzed a commercial database (IMS Health) to assess the potential for improved prescribing of OPR and other drugs. CDC calculated state rates and measures of variation for OPR, long-acting/extended-release (LA/ER) OPR, high-dose OPR, and benzodiazepines.

**Results:**

In 2012, prescribers wrote 82.5 OPR and 37.6 benzodiazepine prescriptions per 100 persons in the United States. State rates varied 2.7-fold for OPR and 3.7-fold for benzodiazepines. For both OPR and benzodiazepines, rates were higher in the South census region, and three Southern states were two or more standard deviations above the mean. Rates for LA/ER and high-dose OPR were highest in the Northeast. Rates varied 22-fold for one type of OPR, oxymorphone.

**Conclusions:**

Factors accounting for the regional variation are unknown. Such wide variations are unlikely to be attributable to underlying differences in the health status of the population. High rates indicate the need to identify prescribing practices that might not appropriately balance pain relief and patient safety.

**Implications for Public Health:**

State policy makers might reduce the harms associated with abuse of prescription drugs by implementing changes that will make the prescribing of these drugs more cautious and more consistent with clinical recommendations.

## Introduction

Persons in the United States consume opioid pain relievers (OPR) at a greater rate than any other nation. They consume twice as much per capita as the second ranking nation, Canada (1). Overprescribing of opioid pain relievers can result in multiple adverse health outcomes, including fatal overdoses (2). Opioid pain relievers were involved in 16,917 overdose deaths in 2011; in 31% of these deaths, benzodiazepine sedatives were also cited as contributing causes (CDC WONDER, unpublished data, 2014). High rates of prescribing these controlled substances are important determinants of rates of fatal overdose and drug abuse (3,4). Overall state prescribing rates of OPR vary widely (5). Variation in prescribing rates for higher-risk opioid prescriptions (e.g., those for long-acting or extended-release [LA/ER] formulations) or those for high daily dosage have not been examined. LA/ER OPR are more prone to abuse, and high-dose formulations are more likely to result in overdoses, so they deserve special attention. Benzodiazepines are commonly prescribed in combination with OPR, even though this combination increases the risk for overdose (6). Interstate variation in prescribing rates for benzodiazepines has not been measured.

Information on local prescribing rates can alert authorities to atypical use and can prompt action. Such authorities include state and local health departments, law enforcement agencies, health-care systems, and licensure boards. States have the authority to track prescribing and dispensing and regulate medical practice within their borders. They can influence the rate of prescribing of controlled prescription drugs by various measures. These include passing regulations related to use of state prescription drug monitoring programs and the operation of pain clinics.

## Methods

Data on prescribing in 2012 come from IMS Health’s National Prescription Audit (NPA). NPA provides estimates of the numbers of prescriptions dispensed in each state based on a sample of approximately 57,000 pharmacies, which dispense nearly 80% of the retail prescriptions in the United States. Prescriptions, including refills, dispensed at retail pharmacies and paid for by commercial insurance, Medicaid, Medicare, or cash were included.*

CDC used the numbers of prescriptions and census denominators to calculate prescribing rates for OPR, subtypes of OPR, and benzodiazepines. The OPR category included semisynthetic opioids, such as oxycodone and hydrocodone, and synthetic opioids, such as tramadol. It did not include buprenorphine products used primarily for substance abuse treatment rather than pain, methadone distributed through substance abuse treatment programs, or cough and cold formulations containing opioids. LA/ER OPR were defined as those that should be taken only 2 to 3 times a day, such as methadone, OxyContin, and Opana ER. High-dose OPR were defined as the largest formulations available for each type of OPR that resulted in a total daily dosage of ≥100 morphine milligram equivalents when taken at the usual frequency, for example, every 4–6 hours. Benzodiazepines included alprazolam, clonazepam, clorazepate, diazepam, estazolam, flurazepam, lorazepam, oxazepam, quazepam, temazepam, and triazolam.

CDC calculated prescribing rates per 100 persons for the United States, each census region, and each state. CDC described the distribution of state rates using mean, standard deviation (SD), coefficient of variation (CV) (SD divided by the mean), the interquartile ratio (IQ) (75th percentile rate divided by the 25th percentile rate), and the ratio of the highest/lowest rates. Rates were transformed into multiples of the SD above or below the mean state rate of each drug.

## Results

Prescribers wrote 82.5 OPR prescriptions and 37.6 benzodiazepine prescriptions per 100 persons in the United States in 2012 (Table). LA/ER OPR accounted for 12.5%, and high-dose OPR accounted for 5.1% of the estimated 258.9 million OPR prescriptions written nationwide. Prescribing rates varied widely by state for all drug types. For all OPR combined, the prescribing rate in Alabama was 2.7 times the rate in Hawaii. The high/low ratio was greater for LA/ER OPR and high-dose OPR compared with all OPR together: for high-dose OPR, state rates ranged 4.6-fold (Delaware versus Texas), and for LA/ER OPR, state rates ranged 5.3-fold (Maine versus Texas). State rates ranged 3.7-fold (West Virginia versus Hawaii) for benzodiazepines. For both OPR and benzodiazepines, Alabama, Tennessee, and West Virginia were the three highest-prescribing states. Among the OPR drugs, interstate variation was greatest for oxymorphone (CV = 0.72, IQ = 2.50, high/low = 21.9). OPR prescribing rates correlated with benzodiazepine prescribing rates (r = 0.80; p<0.01).

**FIGURE 1:**
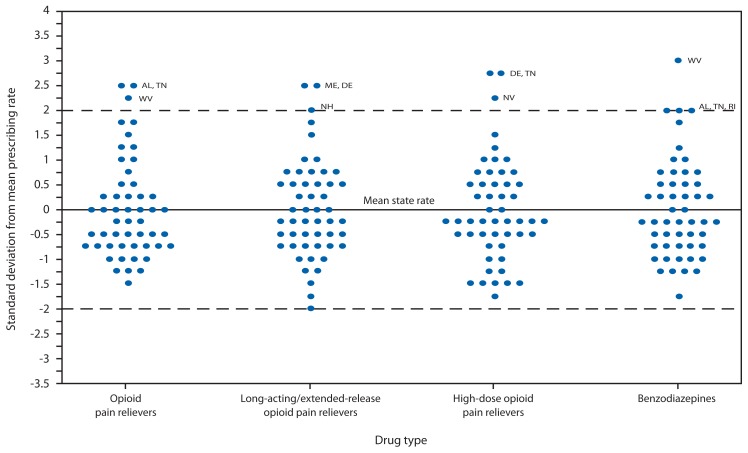
Distribution of state prescribing rates,^*^ by drug type — IMS Health, United States, 2012 * State rates are rounded to the nearest 0.25 standard deviation for purposes of presentation.

The distribution of state prescribing rates was skewed toward higher rates (Figure 1). For both OPR and benzodiazepine rates, Alabama, Tennessee, and West Virginia were ≥2 SDs above the mean. For LA/ER opioids, Maine and Delaware were ≥2 SDs above the mean. For high-dose OPR, Delaware, Tennessee, and Nevada were ≥2 SDs above the mean. Texas’s rate for LA/ER OPR was the only rate ≥2 SDs below the mean for any category.

**FIGURE 2:**
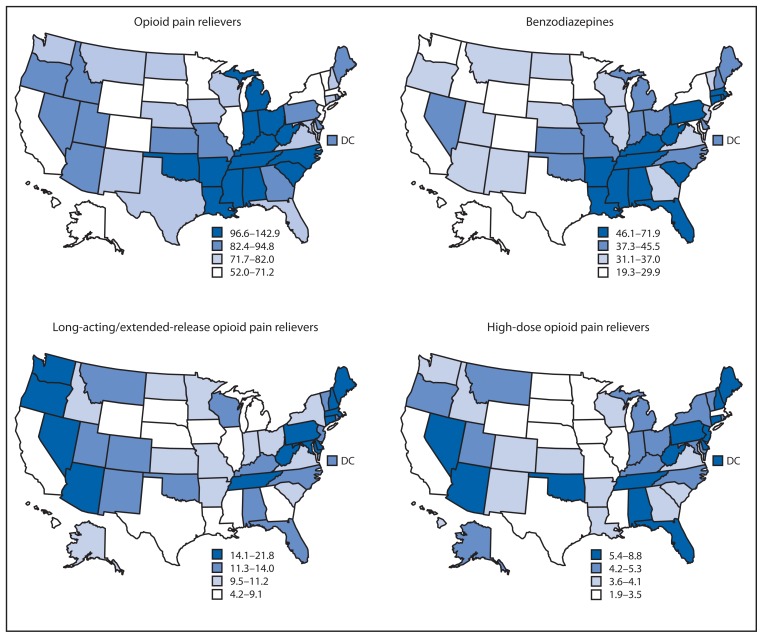
Prescribing rates per 100 persons (in quartiles), by state and drug type — IMS Health, United States, 2012

The South region had the highest rate of prescribing OPR and benzodiazepines (Figure 2). The Northeast had the highest rate for high-dose OPR and LA/ER OPR, although high rates also were observed in individual states in the South and West. In the Northeast, 17.8% of OPR prescribed were LA/ER OPR. States in the South ranked highest for all individual opioids except for hydromorphone, fentanyl, and methadone, for which the highest rates were in Vermont, North Dakota, and Oregon, respectively.

## Conclusions and Comment

The rates of use of pain relievers and benzodiazepine sedatives showed about three- to five-fold variation from the highest to lowest states. Variation was greater for the LA/ER and high-dose formulations of OPR. Higher OPR and benzodiazepine prescribing rates in the South presented in this report are similar to the findings of higher prescribing rates for other drugs in the South, including antibiotics (7), stimulants in children (8), and medications that are high-risk for the elderly (9). Previous studies have found that regional prescribing variation cannot be explained by variation in the prevalence of the conditions treated by these drugs (5,7). Other research indicates that wide variation in rates of surgery and hospitalization also cannot be explained by the underlying health status of the population (9,10). Wide variation in the use of medical technology, including pharmacotherapy, usually indicates a lack of consensus on the appropriateness of its use (9). Therefore, one possible explanation for the results of this study is the lack of consensus among health-care providers on whether and how to use OPR for chronic, noncancer pain (2).

Research on small-area variation in health care indicates that high rates of use of prescription drugs and medical procedures do not necessarily translate into better outcomes or greater patient satisfaction. In fact, high rates of use might produce worse outcomes (11,12). In this case, greater use of opioids and benzodiazepines might expose populations to greater risks for overdose and falls (2,3,13,14). Greater use is also associated with abuse (4), although such use might both cause and be caused by abuse. The wide variation in rates of use for LA/ER opioids, in particular, might reflect the demand for these drugs in the drug-using community and their selective prescribing, often in combination with sedatives and muscle relaxants, by unscrupulous pain clinics (14). Factors that might explain why some states have consistently lower rates of prescribing also need to be identified in future research.

The findings in this report are subject to at least four limitations. First, IMS estimates have not been validated, and they do not include prescriptions dispensed by prescribers, hospital/clinic pharmacies, or health maintenance organization pharmacies, potentially biasing rates downward. Second, prescriptions might be dispensed to nonstate residents, as commonly occurred in Florida during the previous decade (14). Third, prescribing rates cannot be correlated with rates of outcomes, such as overdoses with these drugs, because drug-specific overdose data are not available for most jurisdictions. Finally, the prescribing rates shown for a state might conceal large differences in rates within the state (15).

Evaluating and modifying state prescribing patterns is particularly important in states with the highest prescribing rates for drugs prone to abuse. States can determine the factors driving their high prescribing rates by using data from their prescription drug monitoring programs (PDMPs), systems that record all prescriptions for drugs prone to abuse. They can also use PDMPs to evaluate the impacts of policy changes. Recently, a few states have been able to change prescribing patterns by increasing prescriber use of their PDMPs. New York and Tennessee, for example, mandated prescriber use of the state PDMP in 2012. They subsequently used their PDMPs to document declines of 75% and 36%, respectively, in the inappropriate use of multiple prescribers by patients (16).

**Key Points**

- Opioid pain relievers and benzodiazepine sedatives are commonly prescribed in the United States. They are frequently prescribed to the same patient.
- Overprescribing of opioid pain relievers can result in multiple adverse health outcomes, including fatal overdoses.
- Wide variation exists from one state to another in prescribing rates for these drugs. For states that prescribe well above the national rate, the need for a change in prescribing practices is urgent.
- CDC recommends that states make active use of their prescription drug monitoring programs to calculate current rates of prescribing, examine variations within the state, and track the impact of safer prescribing initiatives.
- Additional information is available at http://www.cdc.gov/vitalsigns.

States can take other actions that will affect prescribers. Developing or adopting existing guidelines for prescribing OPR and other controlled substances can establish local standards of care that might help bring prescribing rates more in line with current best practices. State Medicaid programs can manage pharmacy benefits so as to promote cautious, consistent use of OPR and benzodiazepines. In addition, a number of states have passed laws designed to address the most egregious prescribing excesses. Florida, for example, enacted pain clinic legislation in 2010 and prohibited dispensing by prescribers in 2011. It subsequently experienced a decline in rates of drug diversion (17) and a 52% decline in its oxycodone overdose death rate (18). Guidelines, insurance strategies, and laws are promising interventions that need further evaluation. Patients in all states deserve access to safe and effective evidence-based medical care, and prescribers should carefully consider the balance between risks and benefits in any pharmacotherapy.

## Figures and Tables

**TABLE:** Prescribing rates per 100 persons, by state and drug type — IMS Health, United States, 2012

State	Opioid pain relievers	Rank	Long-acting/extended-release opioid pain relievers	Rank	High-dose opioid pain relievers	Rank	Benzodiazepines	Rank
Alabama	142.9	1	12.4	22	6.8	4	61.9	2
Alaska	65.1	46	10.7	31	4.2	26	24.0	50
Arizona	82.4	26	14.5	12	5.5	12	34.3	33
Arkansas	115.8	8	9.6	37	4.1	29	50.8	8
California	57.0	50	5.8	49	3.0	42	25.4	47
Colorado	71.2	40	11.8	24	4.1	31	28.0	44
Connecticut	72.4	38	14.1	13	5.4	13	46.2	11
Delaware	90.8	17	21.7	2	8.8	1	41.5	19
District of Columbia	85.7	23	13.7	17	5.7	10	38.4	24
Florida	72.7	37	11.3	26	6.6	5	46.9	10
Georgia	90.7	18	8.6	43	4.1	30	37.0	27
Hawaii	52.0	51	8.8	42	3.9	36	19.3	51
Idaho	85.6	24	10.3	33	3.9	34	29.1	42
Illinois	67.9	43	5.2	50	2.0	50	34.2	34
Indiana	109.1	9	10.7	30	4.9	20	42.9	17
Iowa	72.8	36	7.3	47	2.2	48	37.3	26
Kansas	93.8	16	10.3	34	4.0	32	38.9	23
Kentucky	128.4	4	11.6	25	5.0	19	57.4	5
Louisiana	118.0	7	7.8	46	3.6	39	51.5	7
Maine	85.1	25	21.8	1	5.6	11	40.7	22
Maryland	74.3	33	16.0	6	5.0	18	29.9	40
Massachusetts	70.8	41	14.9	8	3.5	41	48.8	9
Michigan	107.0	10	9.1	40	4.5	22	45.5	14
Minnesota	61.6	48	10.2	35	2.2	49	24.9	48
Mississippi	120.3	6	7.2	48	2.9	43	46.2	12
Missouri	94.8	14	9.5	38	3.5	40	42.6	18
Montana	82.0	27	14.0	15	4.4	23	33.7	35
Nebraska	79.4	28	7.8	45	2.3	46	35.0	32
Nevada	94.1	15	14.8	10	8.2	3	37.5	25
New Hampshire	71.7	39	19.6	3	6.1	7	41.2	21
New Jersey	62.9	47	11.3	27	5.8	9	36.5	28
New Mexico	73.8	35	12.7	21	3.8	38	31.5	37
New York	59.5	49	9.5	39	4.3	24	27.3	45
North Carolina	96.6	13	13.7	18	4.3	25	45.3	15
North Dakota	74.7	32	10.5	32	2.3	47	31.1	39
Ohio	100.1	12	11.2	28	4.2	27	41.3	20
Oklahoma	127.8	5	12.8	20	6.0	8	44.5	16
Oregon	89.2	20	18.8	4	5.2	16	31.4	38
Pennsylvania	88.2	21	14.9	9	5.4	14	46.1	13
Rhode Island	89.6	19	14.0	14	5.2	17	60.2	4
South Carolina	101.8	11	11.0	29	3.9	33	52.6	6
South Dakota	66.5	45	9.0	41	2.5	45	28.0	43
Tennessee	142.8	2	18.2	5	8.7	2	61.4	3
Texas	74.3	34	4.2	51	1.9	51	29.8	41
Utah	85.8	22	12.1	23	5.3	15	35.9	30
Vermont	67.4	44	13.9	16	4.7	21	35.5	31
Virginia	77.5	29	9.9	36	3.8	37	36.4	29
Washington	77.3	30	14.6	11	4.1	28	27.1	46
West Virginia	137.6	3	15.7	7	6.2	6	71.9	1
Wisconsin	76.1	31	13.1	19	3.9	35	33.4	36
Wyoming	69.6	42	8.0	44	2.7	44	24.1	49
*Mean*	*87.3*	*—*	*12.0*	*—*	*4.5*	*—*	*39.2*	*—*
*Standard deviation*	*22.4*	*—*	*3.9*	*—*	*1.6*	*—*	*11.1*	*—*
*Coefficient of variation*	*0.26*	*—*	*0.32*	*—*	*0.36*	*—*	*0.28*	*—*
*Median*	*82.4*	*—*	*11.3*	*—*	*4.2*	*—*	*37.3*	*—*
*25th percentile*	*71.7*	*—*	*9.5*	*—*	*3.7*	*—*	*31.1*	*—*
*75th percentile*	*96.6*	*—*	*14.1*	*—*	*5.4*	*—*	*46.1*	*—*
*Interquartile ratio*	*1.3*	*—*	*1.5*	*—*	*1.4*	*—*	*1.5*	*—*
*Ratio of highest to lowest*	*2.7*	*—*	*5.3*	*—*	*4.6*	*—*	*3.7*	*—*
Northeast	70.8		12.6		4.8		38.2	
South	93.7		10.2		4.6		43.1	
Midwest	88.4		9.3		3.4		38.1	
West	68.0		9.6		3.9		27.9	
**U.S. rate**	**82.5**	**—**	**10.3**	**—**	**4.2**	**—**	**37.6**	**—**

## Footnotes

* Additional information available at http://www.imshealth.com/deployedfiles/ims/global/content/insights/researchers/npa_data_brief.pdf.
